# Diabetes Increases Cryoinjury Size with Associated Effects on Cx43 Gap Junction Function and Phosphorylation in the Mouse Heart

**DOI:** 10.1155/2016/8789617

**Published:** 2015-12-14

**Authors:** Joseph A. Palatinus, Robert G. Gourdie

**Affiliations:** ^1^Department of Medicine, Beth Israel Deaconess Medical Center, Boston, MA 02215, USA; ^2^Virginia Tech Carilion Research Institute, Roanoke, VA 24016, USA; ^3^Virginia Tech-Wake Forest University School of Biomedical Engineering and Sciences, Blacksburg, VA 24061, USA; ^4^Department of Emergency Medicine, Virginia Tech Carilion School of Medicine, Roanoke, VA 24016, USA

## Abstract

Diabetic patients develop larger myocardial infarctions and have an increased risk of death following a heart attack. The poor response to myocardial injury in the diabetic heart is likely related to the many metabolic derangements from diabetes that create a poor substrate in general for wound healing, response to injury and infection. Studies in rodents have implicated a role for the gap junction protein connexin 43 (Cx43) in regulating the injury response in diabetic skin wounds. In this study, we sought to determine whether diabetes alters Cx43 molecular interactions or intracellular communication in the cryoinjured STZ type I diabetic mouse heart. We found that epicardial cryoinjury size is increased in diabetic mice and this increase is prevented by preinjury insulin administration. Consistent with these findings, we found that intercellular coupling via gap junctions is decreased after insulin administration in diabetic and nondiabetic mice. This decrease in coupling is associated with a concomitant increase in phosphorylation of Cx43 at serine 368, a residue known to decrease channel conductance. Taken together, our results suggest that insulin regulates both gap junction-mediated intercellular communication and injury propagation in the mouse heart.

## 1. Introduction

Diabetic patients are more prone to death following myocardial infarction (MI) [1] independent of coronary vessel patency or left ventricular function [2]. Diabetic patients also exhibit increased infarct size after MI [3]. Infarct size is independently associated with mortality after MI [4], and it is this increase in infarct size that is believed to be the cause of increased mortality in diabetic patients [4]. The STZ diabetic rat exhibits increased infarct size after ligation of the coronary artery compared to nondiabetic controls [5]. This increased necrotic area may reflect either a generalized decreased ability of the diabetic myocardium to heal or an increased susceptibility of the myocardium to injury.

The gap junction (GJ) allows for organized propagation of electrical signals and metabolic coupling between cells. Disruption of GJs is known to be associated with pathological states including arrhythmias in the heart and wound healing disturbances in the skin. Green, Becker, and coworkers reported that knockdown of the gap junction protein connexin 43 (Cx43) by antisense RNA sped wound closure and limited extension of skin burn injuries in rodent models [6, 7]. Cx43 is markedly upregulated in dermal tissues surrounding diabetic foot ulcers and in clinical trials treatment with the Cx43 carboxyl-terminal mimetic peptide *α*CT1 significantly accelerates the rate of closure of these pathologic, slow-healing wounds [8].

Here, we used STZ diabetic mice in conjunction with a previously described cardiac cryoinjury model [9], enabling precise control of the initial magnitude of tissue damage to the ventricle. Consistent with observations in skin, we find that, compared to controls, the extent of myocardial damage is significantly increased in STZ diabetic mice in association with notable changes in the organization of Cx43 GJs and levels of intercellular coupling within ventricular tissues.

## 2. Materials and Methods

### 2.1. Animal Husbandry

Animal care was in accordance with institutional guidelines at the Medical University of South Carolina, Charleston, SC, USA (animal welfare assurance number: A4328-01).

### 2.2. Diabetes Induction

Diabetes was induced in 13-week-old C57/Blk6 mice (Charles River) by an IP injection of 65 mg/kg streptozotocin (Sigma) (STZ) in 0.1 M citrate buffer 1 time per day for 3 days. One week after induction, tail vein glucose was measured using a Accu-Chek Active blood glucose meter (Roche) and mice were considered diabetic if nonfasting glucose was measured to be greater than 250 mg/dL. Animals injected with citrate buffer only, or not injected, served as controls. As an additional control, a group of STZ diabetic mice were injected with 2 units of Lantus insulin (Sanofi Aventis) on alternating days for a 4–6-week time course for cryoinjury studies or a 2–6-week time course for dye coupling studies. To determine insulin effects on injury, insulin was administered 15 minutes to 1 hour before cryoinjury.

In all cases, immediately prior to procedures (either sampling for immunoconfocal, cryoinjury, or cell coupling studies), blood glucose was recorded. Prior to procedures, insulin-treated STZ mice were given additional insulin (up to 5 U) until tail vein glucose was <200 mg/dL. Control mice + insulin were given up to 5 U until tail vein glucose was <100 mg/dL. Control mice, not given STZ, were only utilized if tail vein glucose was <250 mg/dL. Non-insulin-treated diabetic mice were excluded if tail vein glucose was <250 mg/dL. After 4–6 weeks of streptozotocin-induced diabetes, mice exhibited significantly less weight gain and significantly higher tail vein blood glucose and appeared grossly ill.

### 2.3. Nontransmural Cryoinjury

After 4–6 weeks of diabetes, mice underwent a survival surgery to induce nontransmural myocardial cryoinjury using protocols adapted from O'Quinn and others [9]. These mice were prepared for surgery by removing hair on the ventral surface with an electric clipper and disinfecting the area with Betadine antiseptic solution. Under anesthesia (ketamine, 100 mg/kg, Acepromazine, 3 mg/kg, IP, or 5% isofluorane), mice were placed in the supine position and the trachea was intubated with a 22-gauge Angiocath over a blunt introducer. The mice were ventilated with 2% isoflurane in O_2_ at a tidal volume of 250 *μ*L and 150 cycles/minute. At this point, the animals received a subcutaneous injection of the analgesic Carprofen (4 mg/kg) to prevent postoperative pain. Maintaining sterile technique, left thoracotomy was performed in the fourth intercostal space. All muscles overlying the intercostal space were dissected free and retracted with 5-0 silk threads. Only the intercostal muscles were transected. After opening the pericardium, a prechilled cryoprobe (AC3: Brymill Cryogenics with a 3 mm flat surface cryoprobe) was placed against the epicardial surface of the heart for 5 seconds. The cryoprobe was removed and the injury was inspected for uniformity. The incision was closed in layers using 6-0 silk and the skin was sealed with Dermabond veterinary adhesive. The mice were placed on ambient oxygen and allowed to recover until normal respiration was obtained. The animals received one additional Carprofen injection (q 24 hr) before being returned to the housing facility. The animals were sacrificed 48 hours or 1 week after injury. Approximately 15 minutes before sacrifice, mice were administered 1000 units of Heparin via IP injection. In the instance of respiratory distress (shallow, abnormal breathing), the animals were excluded from the study and subsequently euthanized. The method of euthanasia was isoflurane followed by cervical dislocation.

### 2.4. TTC Staining

For the assessment of injury size, hearts were apically perfused with PBS followed by 1% 2,3,5-triphenyltetrazolium chloride (TTC: Sigma) in PBS for 5 minutes. The heart was removed from the animal and placed in a 37°C PBS 1% TTC bath for 10 minutes followed by 4% paraformaldehyde overnight. The hearts were imaged in order to measure epicardial surface area. All images were recorded with a millimetre scale in view to ensure uniform calibration. Epicardial surface area was calculated from images taken using Leica MZ FLIII on digital images using ImageJ (NIH).

#### 2.4.1. TUNEL Staining

As an additional measure of injury size, cryoinjured hearts were sagittally hemisectioned along the base-apex midline of the injury and frozen in optimal tissue cutting media cooled with liquid nitrogen. Sections were fixed in 1% paraformaldehyde for 10 minutes and postfixed in an ice-cold 2 : 1 ethanol-acetic acid solution. Specimens were stained with an ApopTag Fluorescein In Situ Apoptosis Kit (Millipore, S7110). This kit labels dead/dying cells. It is more commonly known as terminal deoxynucleotidyl transferase mediated dUTP nick end labeling (TUNEL). Sections were visualized using an epifluorescent microscope (Leica: DMLB) equipped with 10x objective and a color chilled 3CCD camera (Hamamatsu). TUNEL staining was measured using ImageJ to measure the depth of the entire left ventricle visible by autofluorescence and the depth of TUNEL positive nuclei. The percentage of LV area that was TUNEL positive was calculated.

#### 2.4.2. Measurement of GJ Coupling in the Mouse Heart

This approach was based on Lucifer Yellow dye spread, a well-established method of assaying GJ coupling in cultured cells [10], and further inspired by in vivo adaption of this method for study of skin wound healing in diabetic rats [7]. This novel protocol was as follows. Mice were anesthetized with isofluorane and ventilated as described above. The thorax was opened and the heart was rapidly excised and washed quickly in 37°C oxygenated Tyrodes solution (126.5 mM NaCl, 1.2 mM MgSO_4_, 1.2 mM KH_2_PO_4_, 14.5 mM NaHCO_3_, 2.6 mM KCl, 20 mM CaCl, and 22.1 mM glucose). The apex was transversely hemisectioned with an uncoated razor blade (GEM 62-0167) and snap frozen in liquid nitrogen for western blot analysis. The remaining freshly cut surface of the base was blotted dry with a Kim wipe and the left ventricular region was placed directly on a 5 *μ*L drop of dye solution on a clean Silguard coated surface. The dye solution was prepared fresh and contained 1% Lucifer Yellow (Sigma, L0259-25MG) and 10% Alexa 647 dextran (Invitrogen, d22914) dissolved in PBS (Sigma, P4417). The heart was removed from the dye after precisely 1 minute and washed quickly in 200 mL of PBS before being fixed in 4% paraformaldehyde for at least 10 minutes. After fixation, the hearts were washed twice in PBS and sagittally sliced along the left ventricular wall to expose the region of the heart through which dye had spread. The freshly cut edges were splayed apart on a glass slide and sandwiched below a coverslip. The coverslip was attached to the slide with wax on three sides forming a chamber around the specimen. The chamber was filled with Flurogel-Mount with Tris Buffer (Electron Microscopy Science, 17985-10) as an antifade reagent, and the 4th side of the chamber was then covered with wax to seal the specimen under the glass coverslip within the chamber. Hearts were imaged using a Leica SP5 laser scanning confocal microscope. Two two-channel single optical sections were recorded per heart in the Alexa 488 and Alexa 633 channels.

#### 2.4.3. Western Blotting

The hearts were homogenized and lysed in 50 mm Tris-HCl 1% NP-40, 150 mM NaCl, 2 mM EGTA, 0.05 M NaF, 100 uM NaVO_4_ PMSF, and complete protease inhibitors. The samples were syringed thrice using a 22-gauge needle over ice and incubated at 4°C with rotation for 30 minutes. Protein concentration was determined using a BCA kit and samples were diluted to 1.5 mg/mL. Samples in 2x loading buffer were resolved in parallel on 12.5% Tris-HCl gels (Criteron XT 345-0014, Bio-Rad) in 1x Tris-Glycine Running buffer (Bio-Rad). Resolved proteins were transferred to Immobilon PVDF at 15 V for 25 min (15 mM Tris, 192 mM glycine, 10% vol/vol methanol, and 0.01% SDS). The membranes were blocked in 5% nonfat dry milk/TBS-Tween-20 and probed with primary antibodies. Subsequently, blots were probed with goat anti-rabbit alkaline phosphatase secondary antibody (1 : 10,000; 4010-04; Southern Biotechnology). Immunoreactive proteins were visualized by chemiluminescence using CDP STAR (Applied Biosystems, T2306) as a substrate. Primary antibodies for Cx43 blotting included S368 Cx43 (rabbit, Cell Signalling, 1 : 1000), S262 Cx43 (rabbit, Santa Cruz, 1 : 500), and total Cx43 (rabbit, Sigma, 1 : 2000).

#### 2.4.4. Statistical Analyses

Statistics and graphing were carried out using Microsoft Excel or GraphPad Prism. Unless otherwise noted, means were compared using Student's *t*-test; *p* values < 0.05 were rejected as not significant. When appropriate, analysis of variance with posttesting was used for multiple comparisons. Data are shown with *p* values as means ± SE.

## 3. Results

### 3.1. STZ-Induced Diabetes Increases Mouse Cryoinjury Size

**Figure 1:**
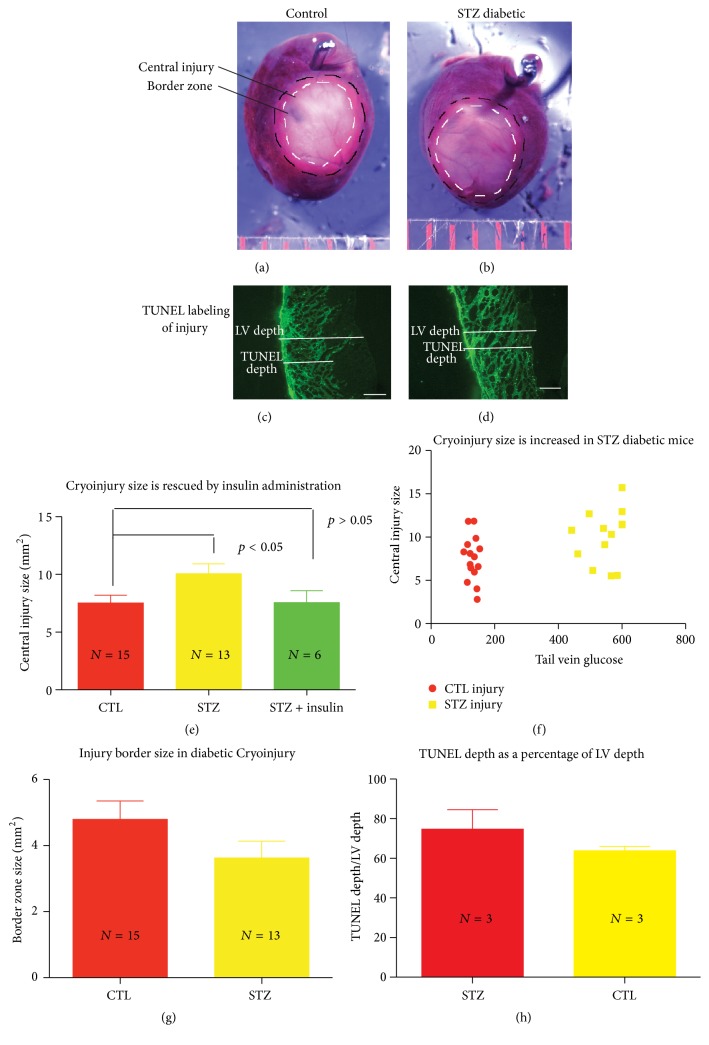
Cryoinjury size is increased in the STZ diabetic mouse. TTC stained control (a) and STZ diabetic (b) rat hearts 48 hrs after cryoinjury. TUNEL labeling of sections from control (c) and STZ diabetic (d) rat ventricles 48 hrs after cryoinjury. Epicardial injury area is increased in the STZ diabetic rodent hearts 48 hrs after cryoinjury and this change is rescued by administration of insulin (e, f). There are trends of decreased width of injury border zone (g) and increased TUNEL labeling (h) in the diabetic heart compared to controls. Spaced bars on (a) = 1 mm. Scale Bar (c, d) = 500 *μ*M.

Cryoinjuries were performed at 4–6 weeks of streptozotocin-induced diabetes. Mice were sacrificed 48 hours after injury and hearts were stained with TTC. Representative images of control (Figure 1(a)) and STZ diabetic cryoinjury (Figure 1(b)), as well as quantification of epicardial injury sizes (Figures 1(e)–1(h)), are shown in Figure 1. The central white area of necrosis was compared between diabetic and control mice. Diabetic mice given insulin every other day and a bolus 15–45 minutes before cryoinjury served as an additional control. These mice did not exhibit significantly larger injuries than nondiabetic mice, suggesting a protective effect of insulin in inhibiting injury spread in these hearts (Figure 1(e)). An injury border zone was observed at the periphery of the central epicardial injury that demonstrated incomplete TTC staining (Figures 1(a) and 1(b)). The pink color observed in this region suggested the presence of both viable and nonviable tissue in this zone. Interestingly, there was a trend towards a decrease in border zone size in the diabetic hearts (Figure 1(g)), although this relationship was not significant (*p* = 0.13).

TUNEL staining of cryoinjuries was performed to measure the area of apoptotic tissue (Figures 1(c) and 1(d)). However, TUNEL labelling of 48-hour cryoinjuries stained the entire injury (Figures 1(c) and 1(d)), making it difficult to discriminate between truly apoptotic cells and cells necrotic from the cryoinfarction. An interesting trend was observed where the depth of TUNEL labelling appeared greater in the diabetic hearts relative to controls (Figure 1(h)); however, this relationship was not significant (*n* = 3 per group, *p* = 0.3).

### 3.2. Insulin Effects on Control Cryoinjury Size

**Figure 2:**
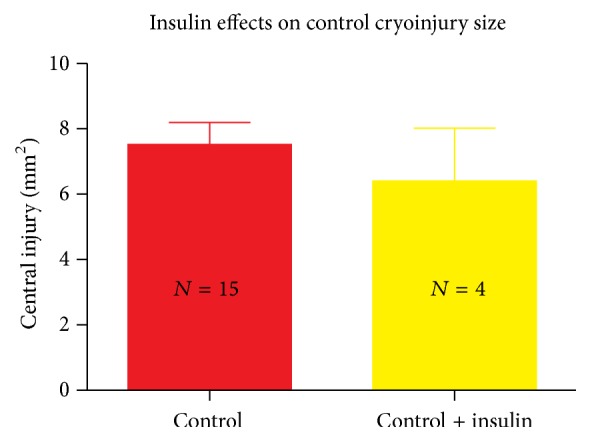
Cryoinjury size is unchanged in the control mouse given insulin 48 hrs following cryoinjury; the epicardial area that does not take up TTC dye is unchanged in the control mouse given insulin.

As lack of insulin was associated with increased cryoinjury size observed in the diabetic mouse, we measured cryoinjury size in the control mice given insulin to induce hypoglycemia (glucose < 100 mg/dL). While there was a trend towards decreasing injury size in the insulin-treated mice (Figure 2), we detected no significant further reduction in injury size in the control mice given insulin compared to untreated control mice (*p* = 0.3).

### 3.3. Insulin Reduces Gap Junction-Mediated Intercellular Communication

**Figure 3:**
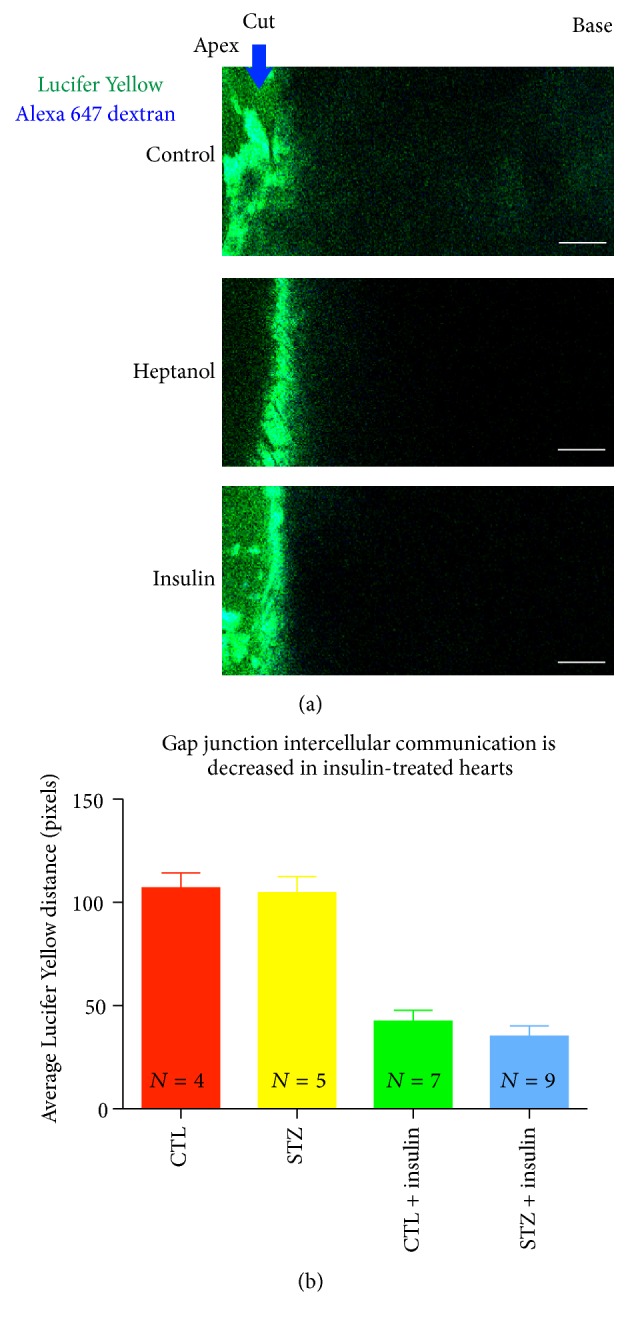
Lucifer Yellow dye spread is decreased in insulin-treated hearts. Representative images of Lucifer Yellow dye propagation through the ventricle of control animals and those treated with heptanol and insulin (a). The propagation of Lucifer Yellow is substantially decreased in hearts treated with insulin independent of STZ treatment (b), *p* < 0.05. Scale bar = 100 *μ*M. Multiple comparisons performed using Kruskal-Wallis test, *p* = 0.0007, applying Dunn's post hoc testing; CTL versus STZ = ns, CTL versus CTL + insulin, and STZ versus STZ + insulin were significantly different.

Because insulin reduces cryoinjury spread in the diabetic heart, we next sought to determine whether intercellular coupling by GJs was associated with the increase in injury size in this model. We measured intercellular coupling by exposing freshly cut hearts to a solution containing the GJ-permeant dye, Lucifer Yellow, and the GJ impermeant dye, Alexa 647 dextran. The results are presented in Figure 3.

Control hearts and diabetic hearts exhibited similar distances of Lucifer Yellow dye spread, which was significantly reduced in the presence of the GJ uncoupler, heptanol. Treatment of control or STZ mice with insulin over the course of 1 week and provision of a bolus dose prior to cardiac excision to induce hypoglycemia (glucose < 100 mg/dL) resulted in a significant decrease in Lucifer Yellow dye spread throughout the ventricle (*p* < 0.03), as assessed by measuring the length of Lucifer Yellow dye spread from the site of furthest Alexa 647 spread (Figure 3).

### 3.4. Insulin Increases Serine 368 Phosphorylation in the Rodent Heart

**Figure 4:**
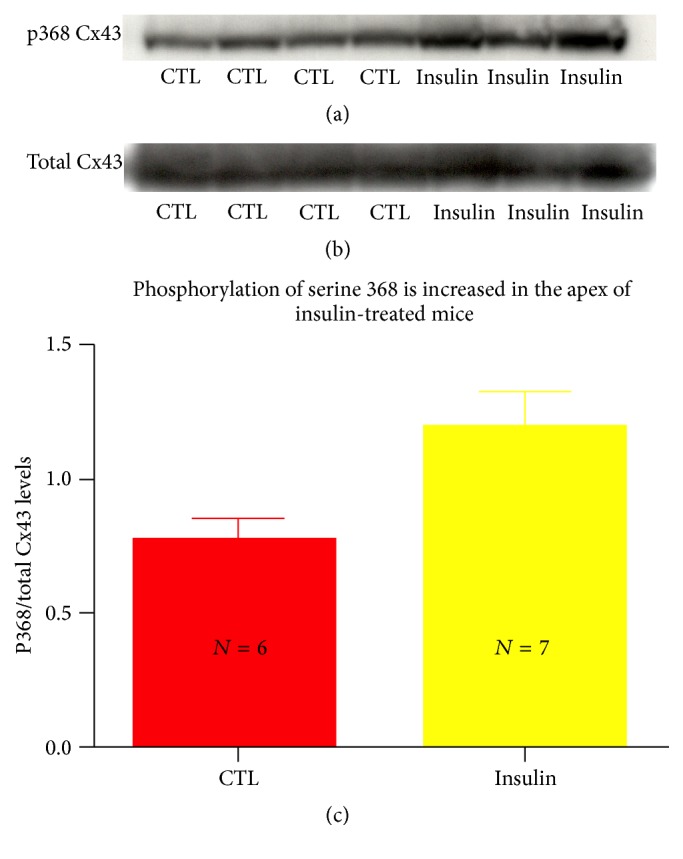
Cx43 phosphorylation at serine 368 is enhanced with insulin administration in the mouse heart. Representative western blot labeling Cx43 phosphorylated at serine 368 (a) and total Cx43 (b) of homogenized ventricles of control mice and those treated with insulin. A significant increase in serine 368/total Cx43 was detected in hearts harvested from insulin-treated animals, *p* < 0.05 (c).

Previous reports have implicated the Cx43 C-terminus in regulating Cx43 channel function. Specifically, Lampe and coworkers demonstrated that phosphorylation of Cx43 at serine at its 368th amino acid residue (serine 368) resulted in a 2-fold reduction of channel conductance [11]. Because decreased GJ communication was detected in response to insulin, we sought to determine whether serine 368 phosphorylation was increased in the insulin-treated mouse heart. The apices of the insulin-treated hearts were homogenized and western blotted for phosphorylated Cx43 p368 and total Cx43. A significant increase in p368 Cx43/total Cx43 in insulin-treated mouse apices was found (*p* < 0.05) (Figure 4). A similar increase in serine 368 levels was observed in diabetic mice given insulin; however, insulin-naïve diabetic mice demonstrated no detectable change in serine 368 levels (data not shown).

### 3.5. Diabetes Increases Levels of Phosphorylation of Cx43 at Serine 262

**Figure 5:**
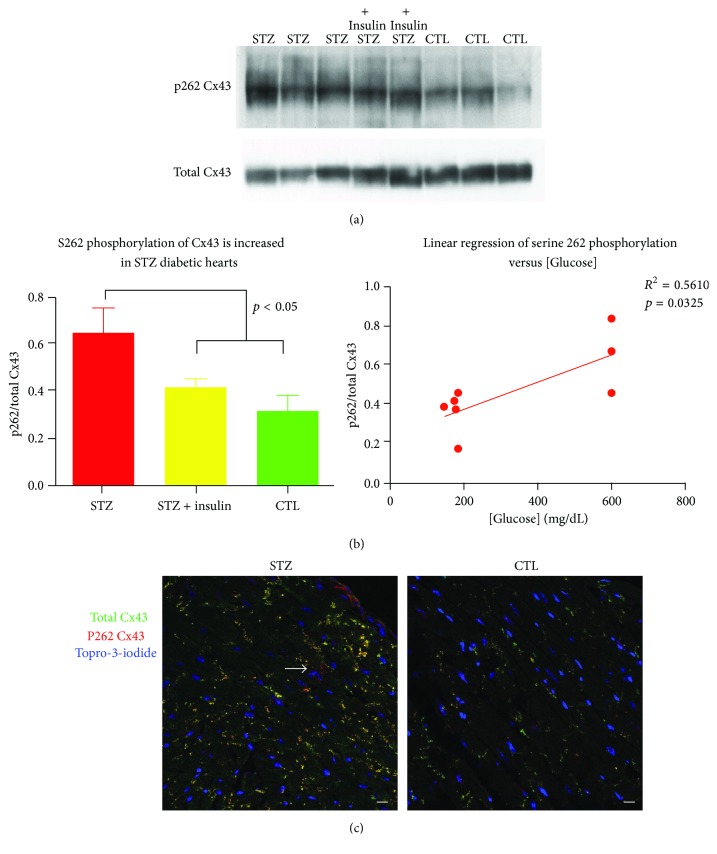
Cx43 phosphorylation at serine 262 is enhanced in the STZ-treated diabetic heart. Representative western blot (a) labeling serine 262 and total Cx43 of the tissue homogenates of ventricular apices harvested from STZ-treated mice, insulin-treated STZ mice, and control mice. (b) Serine 262 phosphorylated Cx43/total Cx43 ratio is significantly increased in STZ-treated mice, *p* < 0.05 (left), and serine 262 phosphorylation/total Cx43 ratio correlates with blood glucose level in diabetic animal (right). (c) Representative confocal images of immunolabeling of STZ and control mice for phosphorylated serine 262 (red) and total Cx43 (green) demonstrating increased serine 262 labeling in the STZ mouse. Arrow indicates perinuclear (nonjunctional) p262 labeling. Scale bar = 10 *μ*M.

Previous reports have indicated that serine 368 and serine 262 phosphorylation are both mediated by protein kinase C. Phosphorylation at these residues is associated with a cardiac injury-resistant state [12]. Given that insulin increases serine 368 phosphorylation, we sought to determine whether insulin and/or diabetes exerted similar effects on serine 262 phosphorylation. In contrast to previous reports in nondiabetic animals, western blotting of serine 262 phosphorylated Cx43 was substantially enhanced in the apices of STZ diabetic mice (Figure 5(a), *p* < 0.05). Administration of insulin reduced serine 262 phosphorylation in these animals, suggesting that the increase was due to the hypoinsulinemia of diabetes and not an effect of the streptozotocin (Figure 5(b), left). A linear regression analysis of p262 Cx43 versus tail vein glucose demonstrated a significant correlation between serine 262/total Cx43 levels and blood glucose (*R* ^2^ = 0.516, *p* = 0.0325) (Figure 5(b), right). Immunofluorescence analysis of STZ diabetic and control injured hearts confirmed an increase in serine 262 phosphorylated Cx43 immunolabeling in these hearts (Figure 5(c)).

## 4. Discussion

Here, we report a number of novel findings: (1) STZ diabetes increases the size of an epicardial cryoinfarction 48 hours after injury, in association with a trend in levels of GJ-mediated intercellular coupling. The increased injury size can be effectively rescued by insulin administration, suggesting that the cause of the increased injury size is due to hypoinsulinemia. (2) Insulin administration also reduces intercellular coupling in both diabetic and nondiabetic animals, suggesting that insulin induces uncoupling of GJs. (3) Insulin administration is associated with increased levels of serine 368 phosphorylated Cx43. Serine 368 is a residue known to be associated with reduced GJ coupling [11] and, consistent with the results presented herein, its phosphorylation in response to insulin has recently been reported in vitro [13] and thus far has not been reported in vivo.

Our observation that insulin reduces cryoinjury size in STZ diabetic mice is consistent with findings in other models of cardiac injury. Marfella and colleagues demonstrated that infarct size in the STZ diabetic mouse is increased in response to ischemia-reperfusion injury [14]. Previous studies examining diabetic injury susceptibility have been controversial [15–17]. But the clinical evidence is clear in that the diabetic heart is particularly sensitive to injury and that hyperglycemia, regardless of whether or not a diagnosis of diabetes has been made, is predictive of cardiac death [18]. MRI quantification studies of infarct size in patients with diabetes or dysglycemia presenting with acute myocardial infarction (MI) demonstrated that blood glucose levels were highly predictive of infarct size [3]. It is long established that diabetic patients are at higher risk of death after a MI. It follows that the goal for treating dysglycemic patients in the setting of MI should be to reduce infarct size. Regulating GJ communication appears to be one way to achieve that goal.

We demonstrate that insulin-induced hypoglycemia significantly reduced GJ-mediated intercellular transfer of Lucifer Yellow. The role of glucose in regulating GJ coupling is complex. Becker's group reported that Cx43 was aberrantly upregulated in the dermis of diabetic skin and that GJ coupling in the diabetic skin was increased. Treatment of diabetic skin with Cx43 antisense oligodeoxynucleotide reduced GJ dye spread and increased the rate of reepithelialization of these wounds [7]. In contrast to the aforementioned findings in diabetic skin, we detected no increase in cell coupling in the setting of diabetic heart (Figure 3). These results may be explained by differences in Cx43 expression between skin and heart. The heart expresses large amounts of Cx43 [19–22], compared to the dermis of skin [7]. It may be that the modest increases in coupling that have been reported from studies of diabetic dermal tissues are not observable in ventricular myocardium of STZ rodents, owing to the high reserve of Cx43-mediated coupling occurring between myocardial cells. Also, consistent with our findings using chemical cell-cell coupling assays, Nygren and colleagues detected no discernable difference in electrical coupling by cardiac GJs in the STZ diabetic rat heart. In the aforementioned study, conduction differences between diabetic and control hearts were only observed in the presence of GJ uncoupler [23]. The resolution of our Lucifer Yellow dye spread assay is likely not sensitive enough to detect this minor difference in coupling.

The finding that insulin reduces GJ coupling in the rodent heart is consistent with reports by Homma and coworkers [24] that demonstrated that insulin induces GJ channel closure in* Xenopus* oocytes. The effect of insulin on channel closure could be eliminated with the deletion of the CT of Cx43 at amino acid 258 and restored by separately expressing portions of the Cx43 CT [24]. Importantly, Homma and coworkers reported that deletion of amino acids 261–280 prevented insulin-induced closure of GJs in this model and coexpression of this sequence was sufficient to restore channel sensitivity to insulin. Here, we report that phosphorylation of Cx43 at serine 262 in the mouse heart correlates directly with tail vein glucose. Taken together with the report of Homma et al. [24], one possibility is that insulin-induced closure of GJs involves this residue. Further supporting a role for serine 262 in regulating cell-cell dye propagation, Doble and others demonstrated that an alanine mutation of serine 262 of Cx43 resulted in increased dye propagation in cultured rat cardiomyocytes [25]. This mutation however did not induce channel closure by the PKC activator PMA. Mutation of other residues, including serine 368, has been reported to prevent GJ closure in response to PKC [11]. In diabetes, PKC-*ε* expression is elevated and Cx43 phosphorylation by PKC-*ε* is increased [26], consistent with the increase in serine 262 phosphorylation that we observe here.

Interestingly, we found that insulin administration increased levels of Cx43 serine 368 phosphorylation in the mouse heart. Homma and coworkers were unable to restore the effects of insulin on GJ closure with a peptide construct containing serine 368 in* Xenopus* oocytes [24]. Differential effects of insulin in* Xenopus* oocytes and mammalian cardiomyocytes may explain these differing results. Insulin translocates the epsilon isoform of protein kinase C (PKC-*ε*) from the cytosol to the membrane of myocytes in vitro [27]. This effect occurs within 2 minutes and lasts over 20 minutes. The time course is consistent with our observation of phosphorylation of Cx43 in response to insulin administration in mouse hearts that received insulin immediately prior to sacrifice. Isoforms of PKC are known to interact with Cx43 at its CT and specifically phosphorylate serines 368 and 262 in mammalian tissues [25, 28]. Additionally, serine 368 phosphorylation is associated with a reduction in channel conductance [29] and single channel permeability, results in agreement with our finding of reduced GJ-mediated coupling in the mouse heart.

The above points being raised, there are caveats to interpretation that should be made. Here, we demonstrate that cryoinfarction produces larger injuries in diabetic mouse hearts, and, in turn, these are reduced by insulin pretreatment. Additionally, we demonstrate that insulin administration increases serine 368 phosphorylation and this is associated with reduced cell coupling. However, it is well established that the effects of insulin are complex and multiform. Insulin is an anabolic hormone involved in numerous signaling pathways and most importantly it regulates metabolic processes in the cell. Insulin and IGF effectors have been shown to enhance mitochondrial metabolism and be cardioprotective in the setting of cardiac failure [30]. It is possible that the increased injury size found in STZ diabetic mice is partially attributable to mitochondrial dysfunction that is acutely recovered by insulin treatment. Additionally, because of multiple anabolic effects of insulin on protein expression, there is concern that insulin administration over the course of the week prior to sacrifice may have exhibited effects on Cx43 expression in this setting. Previous reports have documented that knockdown of greater than 95% of Cx43 is required prior to observance of conduction slowing [31], which was not observed here. We observed no clear difference in Cx43 expression level; however, further work is needed to assess the effect of insulin administration on Cx43 expression.

Further work is also needed to address how serine 368 phosphorylation of Cx43 is regulated and to define its role in the normal and diabetic heart in response to insulin and other cardiac modulating agents. Early reports by Lin and others demonstrated an increase in Cx43 phosphorylation (as identified by different molecular weight bands via immunoblotting) in the diabetic rodent [26]. We expand on these findings here by characterizing phosphospecific isoforms of Cx43 in the diabetic heart. We found that Cx43 phosphorylated at serine 368 was not increased in the diabetic heart but in fact decreased and determined that phosphorylation at this site was increased by insulin administration. The additional site, serine 262 which is also phosphorylated by PKC-*ε*, exhibits increased phosphorylation in diabetes and may be the residue responsible for the shift in Cx43 molecular mass noted in the report of Lin et al. [26].

One tempting hypothesis that stems from our findings is that differential phosphorylation of the Cx43 CT regulates its role in the response to injury. In diabetes, PKC-*ε* activity is increased, but only serine 262 phosphorylation is increased. The phosphostatuses of serine 262 and 368 residues typically mirror each other [32]. The disparity in phosphorylation observed in diabetes suggests that conformational changes within the Cx43 CT, or its interaction with other binding partners, may block the ability of PKC-*ε* to efficiently phosphorylate Cx43 at S368 in the setting of the non-insulin-treated diabetic heart.

## 5. Conclusion

In this study, we demonstrated that an increased area of cell death occurs after cryoinjury in the diabetic mouse heart and that this increase was reduced by a preinjury treatment with insulin. Insulin administration was associated with increased levels of serine 368 phosphorylation of Cx43 and decreases in GJ-mediated intercellular coupling. Additionally, we reported that diabetes results in increases in serine 262 phosphorylation of Cx43. We conclude that insulin is cardioprotective against cryoinjury in our mouse model and that this protection is associated with decreased cell-to-cell coupling. These findings suggest that glycemic control prior to a planned cardiac injury (i.e., cardiac surgery) may assist in the preservation of myocardium. Furthermore, modulation of gap junction-mediated intercellular communication may be a potential therapeutic target for myocardial injury preservation strategies. This being said, further work in humans is needed to evaluate the safety and efficacy of this approach.
